# GMC training survey and missing trainees in psychiatry

**DOI:** 10.1192/bjo.2021.433

**Published:** 2021-06-18

**Authors:** Martin Schmidt, Timothy Leung

**Affiliations:** 1 Surrey and Borders Partnership NHS Foundation Trust; 2 East London NHS Foundation Trust

## Abstract

**Aims:**

To investigate the extent of misattributed responses in the General Medical Council (GMC) National Training Surveys (NTS).

**Background:**

As part of its role in quality assurance of medical training, the GMC conducts an annual survey of trainers and trainees. Benchmarking of trusts’ performance is indicated by red flags denoting outlying poor performance. The validity of this depends on the correct attribution of responses to trusts. We have previously found that responses for Foundation Year One (FY1) trainees undertaking psychiatry placements were misattributed to trainees’ affiliated acute trusts (AT), even though the mental health trusts (MHT) were providing the training placements.

**Method:**

Data from the online reporting tool were used to calculate the numbers of FY1, Foundation Year Two (FY2), and General Practice Speciality trainees (GPST) on psychiatry placements attributed to ATs and MHTs in 2019. A range is provided for the data, as results for trusts with one or two trainees are not reported. The data were analysed by training level and the 13 Health Education England (HEE) regions to give a proportion of trainees missing from the MHT data (% missing), an indication of response misattribution.

**Result:**

296-302 FY1s were attributed to MHTs and 114-148 to ATs, giving a % missing of 27.4-33.3%. 261-275 FY2s were attributed to MHTs and 89-125 to ATs, giving a % missing of 24.4-30.0%. 507-511 GPSTs were attributed to MHTs and 49-73 to ATs, giving a % missing of 8.8-12.6%.

Across the three training levels, all HEE regions were affected by data misattribution. The regions most affected were South London, Kent Surrey Sussex, and North West London, with missing % of 51.6-54.3%, 33.9-40.7% and 29.9-32.5% respectively. The HEE regions least affected were East Midlands, North Central and East London, and East of England, with missing % of 4.3-6.0%, 5.6-8.1% and 5.5-10.4% respectively.

**Conclusion:**

Response misattribution for psychiatry placements in the NTS is rife, with the greatest impact on FY1s. While this issue affects all HEE regions, wide variation exists. Response misattribution means that the calculation of outliers is based on incomplete data, threatening the validity of the results. By liaising with our local HEE office to ensure correct attribution of our trainees, Surrey and Borders Partnership NHS Foundation Trust reduced our % missing from 50.0-56.8% in 2018 to 5.4-10.1% in 2019, thus proving that it is possible to remedy the situation on a local level.

